# Genomic Profiling of Advanced-Stage Oral Cancers Reveals Chromosome 11q Alterations as Markers of Poor Clinical Outcome

**DOI:** 10.1371/journal.pone.0017250

**Published:** 2011-02-28

**Authors:** Srikant Ambatipudi, Moritz Gerstung, Ravindra Gowda, Prathamesh Pai, Anita M. Borges, Alejandro A. Schäffer, Niko Beerenwinkel, Manoj B. Mahimkar

**Affiliations:** 1 Tata Memorial Centre (TMC), Advanced Centre for Treatment, Research and Education in Cancer (ACTREC), Cancer Research Institute (CRI), Navi Mumbai, India; 2 Department of Biosystems Science and Engineering, ETH Zurich, Basel, Switzerland; 3 Swiss Institute of Bioinformatics (SIB), Lausanne, Switzerland; 4 Head and Neck Unit, Tata Memorial Hospital, Tata Memorial Centre (TMC), Mumbai, India; 5 Department of Pathology and Laboratory Medicine, S. L. Raheja Hospital, Mumbai, India; 6 Computational Biology Branch, National Center for Biotechnology Information, National Institutes of Health (NIH), Department of Health and Human Services (DHHS), Bethesda, Maryland, United States of America; Duke-National University of Singapore Graduate Medical School, Singapore

## Abstract

Identifying oral cancer lesions associated with high risk of relapse and predicting clinical outcome remain challenging questions in clinical practice. Genomic alterations may add prognostic information and indicate biological aggressiveness thereby emphasizing the need for genome-wide profiling of oral cancers. High-resolution array comparative genomic hybridization was performed to delineate the genomic alterations in clinically annotated primary gingivo-buccal complex and tongue cancers (*n* = 60). The specific genomic alterations so identified were evaluated for their potential clinical relevance. Copy-number changes were observed on chromosomal arms with most frequent gains on 3q (60%), 5p (50%), 7p (50%), 8q (73%), 11q13 (47%), 14q11.2 (47%), and 19p13.3 (58%) and losses on 3p14.2 (55%) and 8p (83%). Univariate statistical analysis with correction for multiple testing revealed chromosomal gain of region 11q22.1–q22.2 and losses of 17p13.3 and 11q23–q25 to be associated with loco-regional recurrence (*P = *0.004, *P* = 0.003, and *P = *0.0003) and shorter survival (*P = *0.009, *P* = 0.003, and *P* 0.0001) respectively. The gain of 11q22 and loss of 11q23-q25 were validated by interphase fluorescent in situ hybridization (I-FISH). This study identifies a tractable number of genomic alterations with few underlying genes that may potentially be utilized as biological markers for prognosis and treatment decisions in oral cancers.

## Introduction

Oral squamous cell carcinoma (OSCC) is a major cause of morbidity and mortality worldwide, accounting for more than 275,000 new cases and over 120,000 deaths every year [1]. Although there have been improvements in the therapeutic modalities, OSCC-associated morbidity and mortality remain high and have not changed in over three decades [2]. This lack of improvement in survival indicates that tumor size, lymph node involvement and stage, which are considered as markers of disease aggressiveness, do not sufficiently account for the observed variability in clinical outcomes [3]. Therefore, a comprehensive understanding of the pathological mechanisms of OSCC is needed to complement the existing paradigms in assessing disease aggressiveness and prognosis.

Chromosomal abnormalities are a characteristic attribute of cancer cells and they have been used to define specific disease entities. The advent of genome-wide screening methods such as comparative genomic hybridization (CGH), and, more recently, array CGH (aCGH), have opened up new possibilities to catalogue chromosomal aberrations at high resolution [4], [5]. Many chromosomal aberrations that may harbor oncogenes or tumor suppressor genes have emerged as predictive and prognostic markers for tumors [5], [6]. OSCC is reported to arise through the accumulation of numerous specific chromosomal alterations [2]. Gains mapped on chromosomal arms 3q, 6q, 8q, 9p, 9q, 11p, 11q, 14q, 17q, and 20q and losses mapped on 3p, 4q, 9p, and 18q have suggested putative oncogenes and tumor suppressor genes associated with oral cancer [7]–[15]. Molecular profiles of oral cancers vary throughout the world and are influenced by both aetiological factors and ethnicity, yet no conclusive studies have been reported to date [16], [17]. Most genome-wide studies on OSCC have been carried out on various intra-oral sites that are associated with different aetiological agents. Apart from tobacco and alcohol, human papilloma virus (HPV) infection is a known risk factor for OSCC. HPV-infected oropharyngeal tumors comprise a distinct molecular, clinical, and pathological disease entity with distinct genetic alterations and better prognosis [18]–[20].

Previous studies have revealed certain over- and under-expressed genes in oral cancer. Based on the existing literature, we compiled a list of genes associated with oral cancer, which may be useful in identifying and functionally validating driver genes in the underlying regions of alteration. To date, only one study has examined the clinicopathological association of genomic alterations in a small set of OSCC (*n* = 8) [9]. Therefore, the present study aims at delineating genome-wide copy number alterations (CNAs) in oral cancer and to understand whether these genetic alterations are associated with clinical characteristics and prognosis. The study focuses on advanced-stage cancers of the gingivobuccal complex and tongue, which are associated with tobacco use and were found to be unrelated to HPV infection. We demonstrate the potential of high-resolution genome-wide aCGH for calling chromosomal alterations and identifying genomic lesions associated with high risk of relapse and decreased survival time.

## Materials and Methods

### Tissue sample collection and tumor micro-dissection

The study was approved by the human ethics committee of the Tata Memorial Hospital. Neo-primary tumor samples were obtained from 60 patients undergoing surgery for oral cavity cancers at the Head and Neck Unit and were collected from the tumor tissue repository at Tata Memorial Centre, Mumbai. Patients received neither radiation nor chemotherapy before the surgery. The tumor content in the tissues was assessed on a Hematoxylin and Eosin (H&E)-stained section independently by two pathologists. Tissues with more than 70% tumor content were processed for aCGH. Informed written consent was obtained from all participants of the study.

### DNA Isolation from tissues

DNA was extracted following a standard phenol/chloroform protocol. DNA quantification was performed on Nanodrop-1000 spectrophotometer (NanoDrop Technologies, Wilmington, Delaware) and the quality was assessed by electrophoresis on 0.8% agarose gel. A pool of ethnicity and gender-matched normal DNA was isolated from the peripheral blood lymphocytes of healthy donors (*n* = 10) which was used as reference for aCGH.

### HPV Typing

HPV presence was determined by polymerase chain reaction (PCR) using GP5+/6+ primers [21] following confirmation of amplifiable DNA by Beta-Globin PCR [22]. SiHa DNA for HPV16, HeLa DNA for HPV18 (positive controls), and C33A DNA, SCC074 (negative controls) were included while performing all PCR reactions.

### Array CGH Hybridization

Whole-genome copy number profiling was performed on 105K CGH oligonucleotide arrays (Agilent Technologies, Santa Clara, CA) according to the manufacturer's instructions. Briefly, 4.5 µg of tumor and pooled gender-matched reference DNA were labeled with fluorochromes Cy3 and Cy5, respectively. Labeled samples were purified using the genomic DNA purification module (Agilent Technologies), combined, mixed with human Cot-1 DNA, and denatured at 95°C (Oligo aCGH hybridization kit, Agilent Technologies). The mixture was applied to microarrays and hybridization was performed at 65°C for 40 hours. After hybridization, the microarrays were washed with Oligo aCGH wash buffer followed by drying of slides. After drying, the arrays were scanned using an Agilent Scanner (Agilent Technologies), and log_2_-intensities were extracted from raw microarray image files using the Agilent feature extraction software version 9.0 (Agilent Technologies). The raw aCGH data have been submitted to Gene Expression Omnibus (http://www.ncbi.nlm.nih.gov/geo/) with accession number GSE23831.

### Genome mapping and Human structural variation

Genomic coordinates were standardized to the NCBI build 36 (hg18) assembly of the human genome. Loci of structural and copy number variants were obtained from the Database of Genomic Variants (DGV) version 9 at The Centre for Applied Genomics (TCAG, http://projects.tcag.ca/variation/) [23].

### Data Analysis

Raw aCGH intensity values were normalized using the R package snapCGH [24] and segmented with the circular binary segmentation (CBS) algorithm [25]. Recurrent copy number alterations (CNAs) were called using the RAE method [26]. This algorithm identifies significantly recurring CNAs using a background model of genomic variability and computes an empirical false discovery rate (q-value) for each candidate CNA. Within CNAs, the RAE algorithm also identifies subintervals called “focal regions” that were more common. Thresholds for losses/deletions and gains/amplifications were set adaptively from the distribution of segment heights obtained by the CBS algorithm [25]; the thresholds for deletions and amplifications were more stringent than for losses and gains. RAE distinguishes between a “gain” of at least a single copy and an “amplification” by two or more copies. Similarly, RAE defines a “loss” of a single copy and a homozygous “deletion” of both copies [26].

CNAs were considered significant, if their q-value was smaller than 0.1. Recurrent CNAs were further distinguished from known copy number variants (CNVs) present in DGV. For survival analysis, Cox proportional hazards models were calculated with corresponding p-values from the Wald test. Relapse and death from disease were considered as events for recurrence-free and disease-specific survival, respectively. Associations of CNAs with clinical parameters were tested with Fisher's exact test. All statistical computations were performed in R (www.r-project.org).

### Validation of array CGH results using Fluorescence In-situ Hybridization (FISH)

Chromosome 11q alterations associated with recurrence-free and disease-specific survival revealed by aCGH were confirmed by interphase FISH (I-FISH) using a dual color procedure. The probes were prepared by differentially labeling the region and centromere-specific bacterial artificial chromosome (BAC) clones obtained from the Children's Hospital Oakland Research Institute, BACPAC Resources Center. The specificity of all the BAC clones was confirmed on metaphase target slides (Vysis, CA, USA) before hybridizations. BAC clone RP11-135H8 was used as centromere-specific probe for all FISH experiments on chromosome 11, and served as a hybridization control. The copy number status of chromosomal regions 11q22.1-q22.2 and 11q24.1 was determined by applying probes prepared from clones RP11-90M3 and RP11-696J13 respectively and comparing them with the centromeric control. In addition, gain of 7p12 and amplification of 11q13 were validated using locus-specific BAC clones RP11-339F13 and RP11-300I6, respectively. BAC clone RP11-745J15 was used as centromeric probe for chromosome 7. FISH images were captured under a fluorescence microscope (Axioskop II, Carl Zeiss, Germany) and analyzed using the ISIS imaging software (Metasystems, Germany).

#### Comparison of the identified copy number alterations to published data

Using Entrez PubMed, PubMedCentral, and the Science Citation Index, we compiled a list of studies that reported either gene expression changes or copy number changes associated with oral cancer. We also did a focused search for studies suggesting roles for microRNAs in oral cancer, as this has been a topic of increasing interest recently. Wherever possible, gene names were standardized to the name approved by the HUGO Nomenclature Committee (www.genenames.org) as of June 2010.

## Results

### Demographic and clinicopathological characteristics

Array CGH profiling was done for 60 OSCC patient samples. All patients in the cohort were tobacco habitués and were found to be HPV-negative (Table 1, Figure S1). The mean age of the study cohort was 53 years (range, 31–80 years) with a higher proportion of males (80%). Tumors were predominantly moderately differentiated (60%) and were of locally advanced stages III and IV (92%). The cohort had equal representation of node-positive and node-negative groups. The median follow-up period of patients was 22.7 months. Detailed demographic and clinicopathological data of the study cohort is represented in Table S1.

**Table 1:** Clinicopathological characteristics of sixty oral cancer patients.

Characteristics	No. of patients (%)
**Gender**	
Male	48 (80.0)
Female	12 (20.0)
**Mean Age**	53 (range 31–80)
**Tumor sites**	
GBC*	53 (88.3)
Tongue	7 (11.7)
**Habit Profile**	
Exclusive chewers	44 (73.3)
Exclusive smokers	2 (3.3)
Exclusive drinkers	0 (0.0)
Mix habitués†	14 (23.3)
**Pathological Grade**	
Well	2 (3.3)
Moderate	36 (60.0)
Poor	22 (36.7)
**Pathological Cervical Lymph Node** involvement	
Negative (N0)	30 (50.0)
Positive (N+)	30 (50.0)
**Pathological Stage**	
I & II	5 (8.3)
III & IV	55 (91.7)
**Treatment**	
Surgery only	6 (10.0)
Surgery + RT‡	43 (71.7)
Surgery + RT‡+ CT§	11 (18.3)
**Recurrence Status**	
No recurrence	26 (43.3)
Recurrence	25 (41.7)
Lost to follow-up#/unknown	9 (15.0)
**Clinical outcome**	
Alive with no evidence of disease	25 (41.7)
Dead of disease	22 (36.7)
Alive with disease	3 (5.0)
Dead of other cause	1 (1.7)
Lost to follow-up^#^/unknown	9 (15.0)

*GBC: Gingivobuccal complex;

† Mix habitués: Patients with at least two of the habits smoking, chewing, and drinking;

‡ RT: Radiation Therapy;

§ CT: Chemotherapy;

# Lost to follow-up: Patients who did not attend the clinical check-up sessions after primary treatment and as a result their clinical status (recurrence and survival) could not be ascertained.

### Genomic Aberrations

**Figure 1:**
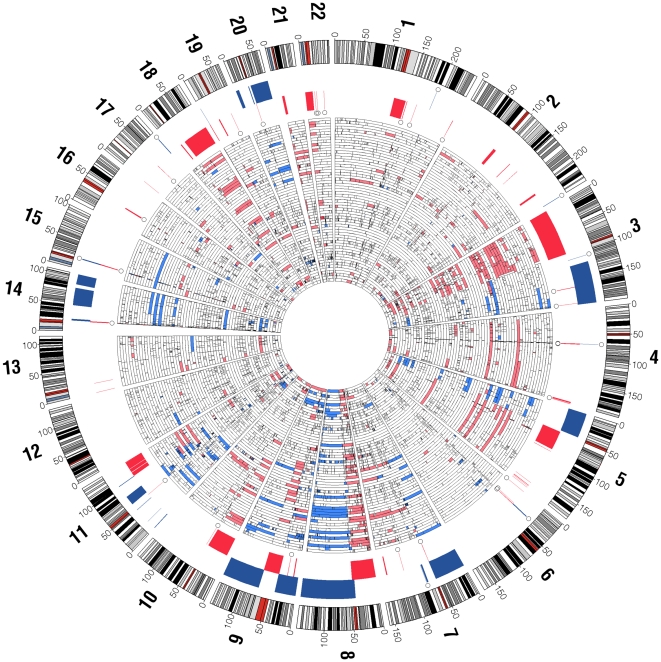
Radial heatmap of recurring copy-number alterations (CNAs) in OSCC. Shown in the inner heatmap are copy number gains/amplifications (blue) and losses/deletions (red), where tumors are stacked radially. Significantly recurring alterations (RAE q-value <0.1) are displayed between the outermost chromosome ideograms and the inner heat map (red: losses, blue: gains). Open circles denote known copy-number variants (CNVs) that span more than 50% with recurring CNAs. Chromosome numbers are shown in bold at periphery of chromosome ideograms with genomic coordinates in megabases.

RAE analysis was performed to identify recurring disease-associated chromosomal aberrations and segregate them from neutral ones. At a false discovery rate of *q* = 0.1, a total of 93 distinct CNAs were found by the RAE algorithm (Figure 1; Figure S2), seven of which in the centromeric regions; for 13 CNAs an additional localized peak region was detected (Table S2). Non-centromeric chromosomal aberrations occurring in more than 20% of the cases are presented in Tables 2 and 3. A large fraction of samples show gross whole chromosome-level alterations (Figure 1). Overall, the number of chromosomal losses (*n = *61, including 7 centromeric) was higher than the number of gains (*n = *32), but the difference was smaller for high-frequency CNAs (*n* = 35 versus *n* = 30). The detailed list of “candidate genes” for all the regions found altered is presented in Table S2.

**Table 2:** Genome-wide alterations in OSCC: Losses & Deletions.

Cytoband	Position		Size (Mb^a^)	q value	% Frequency	
	Start	End			Losses	Deletions
1q11.1-q21.1	120982663	144003083	23.02	8.00E-06	30	5
1p36.33-p11.1	147134204	147499075	0.36	0.000231	23.3	5
1q24.2	167493797	167507911	0.01	0.00029	21.7	13.3
1q44	246713386	246852155	0.14	0.023854	20	10
2q21.2	133504494	133812256	0.31	8.00E-06	25	13.3
3p26.3-p25.3	39095	9150490	9.11	8.00E-06	45	0
3p14.2	60331268	61710321	1.38	8.00E-06	55	18.3
3p26.3-p11.2	39095	95021186	94.98	8.00E-06	61.7	25
3q26.3	163941201	164138371	0.2	8.00E-06	35	30
4q13.2	68901239	69688431	0.79	8.00E-06	43.3	30
4q13.3	70188483	70296201	0.11	8.00E-06	33.3	25
4q35.2	187570439	188216746	0.65	0.026337	20	0
4q35.2	190706301	191176358	0.47	0.000458	25	1.7
5q11.1-q14.3	49759749	88018996	38.26	8.00E-06	36.7	13.3
6p21.34	29962878	29981959	0.02	8.00E-06	26.7	23.3
6p21.33	29962878	29981959	0.02	8.00E-06	26.7	23.3
6p21.32	32605329	32633715	0.03	8.00E-06	43.3	43.3
6p21.32	32519964	32673012	0.15	8.00E-06	43.3	43.3
8p23.2	3400925	4000623	0.6	8.00E-06	65	25
8p11.23	39378080	39464606	0.09	8.00E-06	68.3	36.7
8p23.3-p11.1	63832	47740040	47.68	8.00E-06	83.3	65
9p24.1-p23	8924024	10013871	1.09	2.32E-05	23.3	6.7
9p21.3	21733439	22076827	0.34	8.00E-06	28.3	10
9p13.1-q21.11	38612224	70225195	31.61	8.00E-06	36.7	6.7
10p15.3-p11.1	138235	42150788	42.01	8.00E-06	35	10
10p11.21	37475290	37508431	0.03	8.00E-06	26.7	3.3
10q11.22	46396192	46516611	0.12	2.83E-05	25	5
11q22.3-q23.1	102611683	110151240	7.54	0.018658	20	0
11q23.3-q25	119044645	133316524	14.27	0.0113	20	0
13p13	6365	44752	0.04	8.00E-06	31.7	23.3
15q11.2	18741744	19805989	1.06	2.83E-05	23.3	8.3
18q12.1-q23	24990733	76110993	51.12	8.00E-06	30	5
19p13.3	8275	236875	0.23	8.00E-06	36.7	11.7
22p13-p11	134684	14797037	14.66	0.00964	21.7	5
22q13.1	37689087	37715408	0.03	8.00E-06	25	18.3

The Thresholds for losses of a single copy and homozygous deletions are set adaptively by the RAE method,

a Mb: mega base pair.

**Table 3:** Genome-wide alterations in OSCC: Gains & Amplifications.

Cytoband	Position		Size (Mb^a^)	q value	% Frequency	
	Start	End			Gains	Amplifications
1q31.3	195026732	195104236	0.08	1.04E-05	35.0	28.3
1q31.3	195026732	195048237	0.02	1.04E-05	35.0	28.3
2q37.3	242501268	242717042	0.22	1.81E-05	33.3	21.7
3q13.33-q24	121355348	144551988	23.20	0.001810	21.7	1.7
3q27.1	185019156	185896384	0.88	1.04E-05	48.3	1.7
3q24-q29	145187343	199379595	54.19	1.04E-05	60.0	20.0
4q13.2	69085413	69165843	0.08	1.04E-05	35.0	26.7
5p15.33-p11	75178	46136094	46.06	1.04E-05	50.0	20.0
5p15.33	75178	942987	0.87	1.04E-05	48.3	18.3
6p21.33-p21.32	31524806	32225578	0.70	0.014905	21.7	3.3
6p21.32	32519964	32673012	0.15	4.59E-05	30.0	18.3
7p22.3-p11.1	149297	57562112	57.41	1.04E-05	50.0	20.0
8q24.13-q24.3	123102996	146250794	23.15	1.04E-05	71.7	10.0
8q11.1-q24.4	43452765	146250794	102.80	1.04E-05	73.3	13.3
9p24.3-p21.3	153160	21931457	21.78	1.04E-05	33.3	10.0
9p21.3-p13.1	21980551	39244358	17.26	1.81E-05	38.3	6.7
9q13-q34.3	70238468	140241905	70.00	1.04E-05	40.0	11.7
11q12.2-q14.2	60970713	78077527	17.11	1.04E-05	53.3	26.7
11q13.2-q13.3	68654476	70150073	1.50	1.04E-05	46.7	26.7
11q22.1-q22.2	101407278	102165885	0.76	0.021750	20.0	10.0
14q11.2	19560721	22142166	2.58	1.04E-05	46.7	5.0
14q11.2	21538460	22005864	0.47	1.04E-05	45.0	5.0
14q21.3-q31.1	48265939	80627188	32.36	0.000386	28.3	3.3
14q31.3-q32.33	87604117	106349785	18.75	9.60E-05	38.3	20.0
15q11.2	18741744	20060090	1.32	1.04E-05	35.0	13.3
17q25.3	77385789	78462808	1.08	1.04E-05	28.3	15.0
19p13.3	232109	258746	0.03	1.04E-05	58.3	31.7
20p13-p12.3	18609	5869816	5.85	0.022988	23.3	0.0
20p11.21	24728423	25680524	0.95	0.025337	20.0	0.0
20q11.21-q13.33	29436566	62363603	32.93	1.04E-05	40.0	8.3

The thresholds for gains of a single copy and amplifications by two or more copies were set adaptively by the RAE method.

a Mb: mega base pair.

### Copy number losses

**Figure 2:**
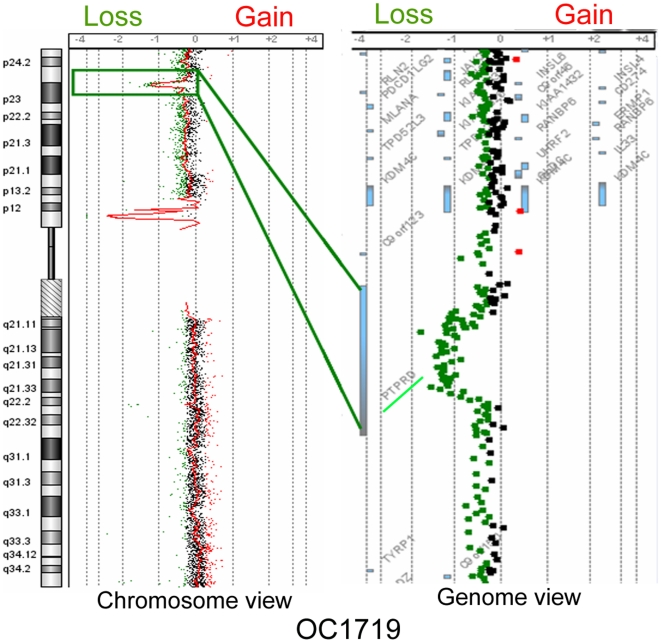
Array CGH based identification of 9p23-p24.1 loss encompassing putative tumor suppressor gene *PTPRD*.

The most frequently occurring losses were identified on chromosomal regions 3p (62%), 5q (37%), 8p (83%), 9p (28%), 10p (35%), 11q (20%), 13p13 (32%), 18q (30%), and 19p12 (13%) as represented in Table S2. Focal regions of loss included 1q24.2 (harboring the candidate gene *NME7*), 2q21.2 (*NCKAP5*), 3p14.2 (*PTPRG*), 3p25.2–p26.3 (*CHL1*, *GRM7*, *RAD18*, *SRGAP3*), 4q35.2 (*MTNR1A*, *FAT1*), 6p21.3 (*HLA-DRA*, *HLA-DRB5*, *HLA-DRB6*, *HLA-DRB1*), 8p23.1 (*CSMD1*), 8p11.2 (*ADAM5P*, *ADAM3A*), 9p21 (*MTAP*, *C9orf53*, *CDKN2A*, *CDKN2BAS*, *CDKN2B*) 9p23-p24.3 (*PTPRD*), 17p13.3 (*RPH3AL*, *MGC70870*), and 22q13.1 (*APOBEC3A*, *APOBEC3B*) and is presented in Table S2. The previously unreported focal loss of 9p23-p24.1 (*PTPRD*) in oral cancer is shown in Figure 2.

### Copy number gains

The most frequent aberrations included gain of chromosomal regions 3q (60%), 5p (50%), 7p (50%), 8q (73%), 9q (40%), 11q13 (47%), 14q (38%), 19p13.3 (58%) and 20q (40%) as represented in Table 3. Focal regions of amplification included 1q31.3 (*CFHR3*, *CFHR1*) 2q37.3 (*LOC728323*) 3q27.1 (*ABCC5*, *ALG3*, *EIF4G1*, *EPHB3*), 5p15.33 (*PDCD6*), 14q11.2, and 19p13.3 (*KIR2 cluster*, *PPAP2C*, *MIER2*) as presented in Table S2.

### Clinicopathological association of chromosomal aberrations

Chromosomal aberrations were analyzed to understand their relevance and associations with clinicopathological parameters like nodal status, grade and clinical outcome. We did not find any significant association of chromosomal aberrations with nodal status or grade. Using the Cox proportional hazards model, we find, at a corrected p-value <0.1, *n* = 11 CNAs associated with recurrence-free and *n* = 12 alterations associated with disease-specific survival (Table 4). Whereas the gains of chromosomal regions 11q12.2-q14.1 (*P* = 0.06) and 11q22.1–q22.2 (*P = *0.009) were associated with poor clinical outcome, gain of 19p13.3 (*P* = 0.04) was associated with better survival. Chromosomal losses of 3p25.3–p26.3 (*P* = 0.08), 6p25.3 (*P* = 0.07), 17p13.3 (*P* = 0.003), 11q23–q25 (*P = *0.0001) and 18p11.1-p11.21 (*P* = 0.04) were associated with poor clinical outcome, while loss of 4q13.2 (*P* = 0.05) was associated with better survival (Table 4).

**Table 4:** Univariate Cox proportional hazards regression analysis of single predictors for recurrence-free and overall survival.

Cytoband	Aberration	Recurrence-free survival		Disease-specific survival	
		BH Corrected p-value*	CPH coef.	BH Corrected p-value*	CPH coef.
3p25.3-p26.3	Loss	>0.1	-	0.08	0.79
4q13.2	Loss	>0.1	-	0.05	−0.55
6p25.3	Loss	0.09	0.52	0.07	0.55
11q12.2-q14.1	Gain	0.06	0.46	0.06	0.48
11q22.1-q22.2	Gain	0.004	0.87	0.009	0.80
11q22.2-q22.3	Loss	0.005	1.25	0.001	1.46
11q23.1-q23.3	Loss	0.004	1.35	0.001	1.51
11q23.3-q25	Loss	0.0003	1.60	0.0001	1.76
11q25	Loss	0.001	1.46	0.0004	1.62
17p13.3	Loss	0.003	1.06	0.003	1.08
18p11.1-p11.21	Loss	0.09	0.78	0.04	0.93
19p13.3	Gain	0.06	−0.51	0.04	−0.59
20q11.21-q13.33	Gain	0.07	0.57	>0.1	-

*Benjamini-Hochberg [49] method of adjusting for multiple tests.

CPH coef.: Cox Proportional Hazard coefficient.

**Figure 3:**
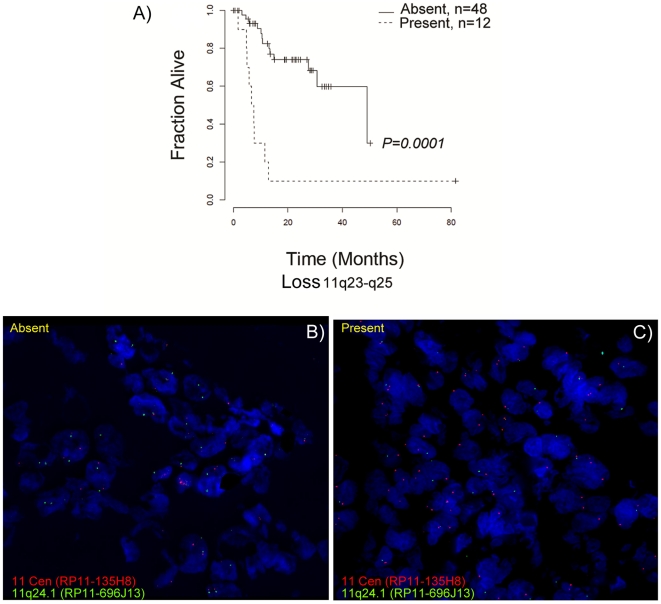
Patient survival curves and FISH validation of 11q23-q25 loss. A) Kaplan-Meier survival estimates of patient groups with and without loss of chromosome 11q23–q25; survival in months (x-axis) is plotted against the fraction of samples alive (y-axis). Interphase FISH analysis detecting the chromosome 11 centromere (red) and the 11q24.1 region (green), B) A case without 11q24.1 loss and C) A case of 11q24.1 loss are shown.

**Figure 4:**
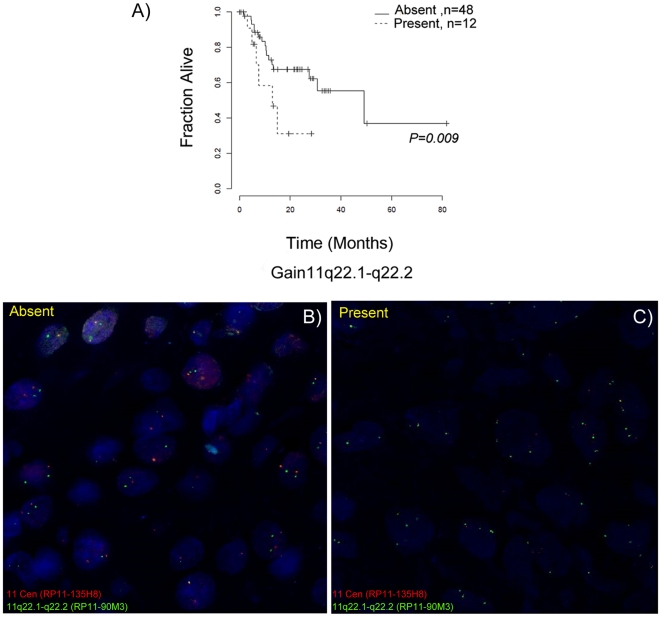
Patient survival curves and FISH validation of 11q22.1-q22.2 gain. A) Kaplan-Meier survival estimates of patient groups with and without gain of chromosome 11q22.1–q22.2; survival in months (x-axis) is plotted against the fraction of samples alive (y-axis). Interphase FISH analysis detecting the chromosome 11 centromere (red) and the 11q22.1–q22.2 region (green), B) A case without 11q22.1-q22.2 gain and C) A case of 11q22.1–q22.2 gain are shown.

Loss of 11q23–q25 (*P = *0.0001) and gain of 11q22.1–q22.2 (*P = *0.009) were found as the strongest predictors of poor clinical outcome in terms of recurrence and survival (Table 4). Kaplan-Meier survival curves for loss of distal 11q and gain of 11q22.1-q22.2 are shown in Figures 3A and 4A. The chromosomal interval 11q23–q25 was subdivided by RAE into four non-overlapping intervals. The *p*-values for the four association tests were almost identical, but the subdivision suggests that there were multiple pertinent genes on 11q23–q25, at least one gene per subinterval.

### Fluorescence in situ hybridization (FISH) analysis

Array CGH results were validated using I-FISH analysis. The samples were selected randomly from the cohort of 60 samples with known array CGH-based copy number alterations. The centromere- (RP11-135H8) and region-specific (RP11-90M3, RP11-696J13) probes hybridized to their target loci showed no cross reactivity (Figure S3). We validated loss of 11q23-q25 (Figure 3B and 3C) and gain of 11q22.1-q22.2 (Figure 4B and 4C) associated with poor clinical outcome and found a concurrence of 70% and 82% respectively with array CGH data. In addition we validated the focal gains 11q13.3 (*CCND1*, *ORAOV1*, *MYEOV*, *FGF3*, *FGF4*, *PPF1A1*, *CTTN*) and 7p12 (*EGFR*) by I-FISH. Results were found to be concordant in 70% and 75% of the samples (Figure S4).

#### Comparison of the identified copy number alterations to published data

We compared the intervals found by RAE (Table S2) to the locations of genes previously suggested in other oral cancer studies. The genes overlapping with each interval are shown in the column ‘OSCC genes’ in Table S2. The functional role of representative candidate genes is discussed.

## Discussion

In this study, we characterized genome-wide alterations in locally advanced, tobacco-associated OSCC to identify markers of poor prognosis for OSCC risk stratification. To our knowledge, this is the first study of OSCC aCGH profiling from the Indian subcontinent. Previous CGH studies of OSCC revealed gains of 8q followed by 3q, 9q, 11q13, 14q, and 20q and losses of 3p followed by 4q, 5q, 8p, 9p, 10q, 11q, 18q, and 21q as the most frequent alterations [7]–[13]. Our study not only validated the previous reports but also revealed novel focal alterations previously not described in oral cancers. Using aCGH, we observed focal gains on chromosomal regions 3q27.1, 5p15.33, 14q11.2 and 19p13.3 and losses on 3p25.2–p26.3 and 9p23-p24.3 (*PTPRD*), which were not reported previously in genome-wide studies of OSCC. The focal alteration of 3q27.1 spans various proto-oncogenes including *ABCC5*, *ALG3*, *EIF4G1* and *EPHB3*. We identified a frequently altered small region on 5p15.33 spanning twenty-four potential oncogenes. These genes, however, do not include *TERT* and *TRIO* that have been proposed in the literature. One of the new candidate gene on this locus is *PDCD6* which has been shown to contribute towards tumor development and expansion [27].

In our study cohort chromosomal arm 3p has been frequently lost, which is consistent with previous reports. We observed a novel focal loss of *RAD18* on chromosome band 3p25.3. RAD18 is an E3 ligase which is reported to play an important role in homologous recombination and repairs double strand break (dsb)[28]. We speculate that loss of *RAD18* may lead to the impaired DNA repair and genomic instability. Our study also reports loss of *PTPRG* on 3p14.2. *PTPRG* encodes receptor-type tyrosine-protein phosphatase gamma acting in growth control by suppressing cyclin D1. A tumor suppressive function of *PTPRG* has been reported in breast cancer [29] and *PTPRG* was one of the earliest suggested oral cancer genes [30] but has not been reported as lost in recent CGH studies. A related member, tyrosine-protein phosphatase delta (*PTPRD*), present on chromosome 9p23-p24.3, was suggested to be gained by Snijders et al. [7], but was not selected as a driver gene for oral cancer. *PTPRD* is a known tumor suppressor for lung cancer [31] and glioblastoma [32]. It antagonizes growth stimulating signaling pathways that are also altered in oral cancers. In our cohort, *PTPRD* had a complex pattern of gains and losses; *PTPRD* was present in a small interval that was lost in 23% of the cases (Table S2), but also in a larger interval of 9p that was gained in 33% of the cases (Table S2). Due to its anti-proliferative function we hypothesize that tumors with *PTPRD* loss may be amenable to therapeutic intervention using growth factor inhibitors.

HPV-related OSCCs are characterized by 16q loss and better clinical outcome. Whereas HPV-unrelated tumors, such as those studied here, had gains of 11q13 and more losses at 3p, 5q, 9p, 15q, and 18q with poor clinical outcome [18]. Array CGH revealed that the samples in this study exhibit a genome-wide profile similar to previously published HPV-unrelated OSCC specimens from other parts of the world, substantiating the presence of a distinct genomic profile of HPV-free OSCCs.

Most OSCC patients report with locally advanced disease at the time of diagnosis (www.seer.cancer.gov/statfacts/html/oralcav.html#survival). The overall survival of these patients is generally poor as the majority of patients develop recurrent disease with chemo- and/or radio-resistance. Patients at similar stages of OSCC, however, do not have identical course of disease and often differ in their clinical outcome. Hence, we analyzed advanced-stage OSCC samples to delineate the genomic alterations that could identify subsets of tumors differing with respect to recurrence and survival. We note that the gain of chromosomal region 11q22.1–q22.2 (*P = *0.009), losses of 17p13.3 (*P* = 0.003) and 11q23–q25 (*P = *0.0001) are associated with poor clinical outcome. These regions were also found to be significantly associated with recurrence-free survival (*P = *0.004, *P* = 0.003, and *P = *0.0003, respectively). Although the association of these chromosomal loci with poor clinical outcome is novel, the loci have been previously reported altered in OSCC [7], [8], [33]. In our study, clinical outcome was strongly associated with specific genomic aberrations detected by aCGH, however, there was no significant association with clinicopathological markers such as nodal status, grade or stage. This finding emphasizes the usefulness of genomic alterations as independent markers of prognosis.

Amplification of 11q13 is reported in about 45% of HNSCC [34], [35]. We find the amplification in 47% of OSCC samples, similar to the earlier reports. The amplification of the 11q13 region was validated using locus-specific FISH. Contradictory reports exist on the association of 11q13 alterations with clinical outcome [36]–[38]. We did not find any significant association of 11q13 with clinicopathological parameters or survival. In the breakage-fusion-bridge (BFB) cycle model of 11q13 amplification, distal 11q loss precedes 11q13 amplification and is therefore considered an early event in HNSCC progression [39]. Jin et al. reported that, in addition to 11q13 amplification, loss of distal 11q may be important for biological aggressiveness of head and neck carcinomas [40]. Further, they found that tumors with 11q loss had concomitant 11q13 amplification. In our cohort only one case (1.7%) had loss of distal 11q without the presence of 11q13 gain (Table S3).

Loss of chromosomal region 11q23-25 was significantly associated with poor clinical outcome. The results so obtained were confirmed by I-FISH. Parikh et al. reported the loss of distal region of 11q in HNSCC cell lines encompassing several DNA damage response encoding genes (*MRE11*, *ATM*, *H2AFX*) and found that this leads to compromised DNA damage response and reduced sensitivity to ionizing radiation [33]. Henson et al. reported a decreased expression of microRNAs miR-125b and miR-100 present on distal 11q in OSCC cell lines and showed their role in the development and progression of disease [41]. These microRNAs were regulated in a copy number dependent fashion as well as via decreased expression of *ATM* [41]. Parikh et al. predicted direct translational relevance for HNSCC patients, as patients with distal 11q loss did not benefit from aggressive radiation therapy [33]. Since in our study tumors with distal 11q loss were found to be a subset of tumors with 11q13 gain, we hypothesize that the distal 11q loss may be used as a risk marker to identify patients who do not benefit from aggressive radiation therapy, but could alternatively benefit from CCND1 inhibitors.

Another predictor of poor survival is the gain of 11q22.1–q22.2. Snijders et al. reported the presence of this rare amplicon in 5.6% of OSCC cases [7]. *YAP1*, *BIRC2* and *MMP7* genes present in this region were proposed as the candidate driver genes based on their role in apoptosis, cell adhesion and migration; *BIRC3* was also mentioned, but not identified as a driver gene. Baldwin et al. reported the copy number gain of 11q22.2–q22.3 amplicon at a higher frequency (15%) and identified two gene clusters with nine matrix metalloproteinase (*MMP*) genes and two baculoviral IAP repeat-containing protein (*BIRC*) genes [8]. In our study, the 11q22.1–q22.2 amplicon encompassing *TRPC6*, *ANGPTL5* and *YAP1* was associated with poor clinical outcome. The frequency of this alteration was 20%, similar to the frequency reported by Baldwin et al. [8].

*YAP1* can itself promote proliferation and transformation or it can act as a transcriptional cofactor by regulating the expression of various transcription factors including *RUNX2*, *SMAD7*, *p73*, *p53BP2* and the TEA domain (*TEAD*) transcription factor family members [42]. *YAP1* can induce anchorage-independent growth, epithelial mesenchymal transition, growth factor-independent proliferation, inhibit apoptosis and activate AKT and ERK pathways [43]. Another candidate gene on 11q22 is *TRPC6*, as suggested by two recent studies which reported overexpression of *TRPC6* in glioma and glioblastoma multiforme (GBM) and analyzed its functional importance in cell growth, proliferation and increased radioresistance [44], [45]. Although the relevance of *TRPC6* function in OSCC needs to be further explored, our data indicate that *TRPC6* may be one of the key genes responsible for radioresistance and poor clinical outcome in OSCC.

We report chromosomal loss of 17p13.3 in 13% of oral cancer samples analyzed. No previous study of oral cancer has identified the loss of this locus. Losses of 17p13.3 have been reported in many solid tumors including lung cancers [46]. The only known gene in the precise region chr17:118,535–134,424 is *RPH3AL*, but there exists no conclusive evidence for a tumor suppressive role [47], [48] despite the fact that loss of 17p13.3 is strongly associated with poor recurrence-free and disease-specific survival.

In summary, our study reports genome-wide alterations in tobacco-associated, HPV-unrelated oral cancers. The study revealed genomic lesions on chromosome arms 11q and 17p13.3 associated with a high risk of relapse and decreased survival. These genomic alterations can potentially help in risk stratification of oral cancer patients beyond the currently used clinical paradigms. Our findings demonstrate the use of genetic alterations for predicting disease outcome, which may be helpful in developing accurate and objective markers for the prognosis of oral cancers.

## Supporting Information

Figure S1 **Screening of HPV DNA in tumor samples.** Representative gel picture of HPV general primer pair (GP5+/6+) PCR. Beta-globin PCR was done to check the genomic integrity in oral cancer samples. CaSki (HPV-16) and HeLa (HPV-18) cervical cell lines were used as positive controls, while C-33A and SCC074 (HPV negative) cervical and oral cell lines were used as negative controls. M: 50 bp marker.
(TIF)

Figure S2 **Manhattan plot for statistically significant genomic alterations in OSCC.** The false discovery rates (*q*-value; y-axis) for gains (blue) and losses (red) are plotted against the 22 autosomes (x-axis). The threshold for significance (*q*≤0.1) is indicated by a dotted line. Stars (*) indicate known copy-number variants (CNVs) according to the database of genomic variants (DGV).
(TIF)

Figure S3 **Metaphase FISH confirming the specificity of BAC clones.** A representative FISH image of metaphase plates with A) FISH probes confirming the specificity of loci 11q22.1-q22.2 (Green signals), B) 11q24.1 (Green signals) and chromosome 11 centromere (Panels A and B, Red signals).
(TIF)

Figure S4 **Interphase FISH validating the gain of 11q13.3 and 7p12 revealed by array CGH.** A) Validation of 11q13.3 amplification by interphase FISH using centromere-specific (Cy3, Red) and locus-specific (FITC, Green) probe. B) Validation of 7p12 amplification by interphase FISH using centromere-specific (Cy3, Red) and locus-specific (FITC, Green) probe.
(TIF)

Table S1 Detailed demographic and clinicopathological data for the study group.
(DOC)

Table S2 A compendium of chromosomal alterations in 60 oral tumors with gene list and known candidate genes cited in the literature.
(XLS)

Table S3 Case-wise chromosomal aberrations at 11q in oral cancer patients.
(DOC)
