# Supplementary figures and images for: Genomic Profiling of Advanced-Stage Oral Cancers Reveals Chromosome 11q Alterations as Markers of Poor Clinical Outcome
Source: PLoS One. 2011 Feb 28;6(2):e17250. doi: 10.1371/journal.pone.0017250 (PMC3046132; doi:10.1371/journal.pone.0017250)

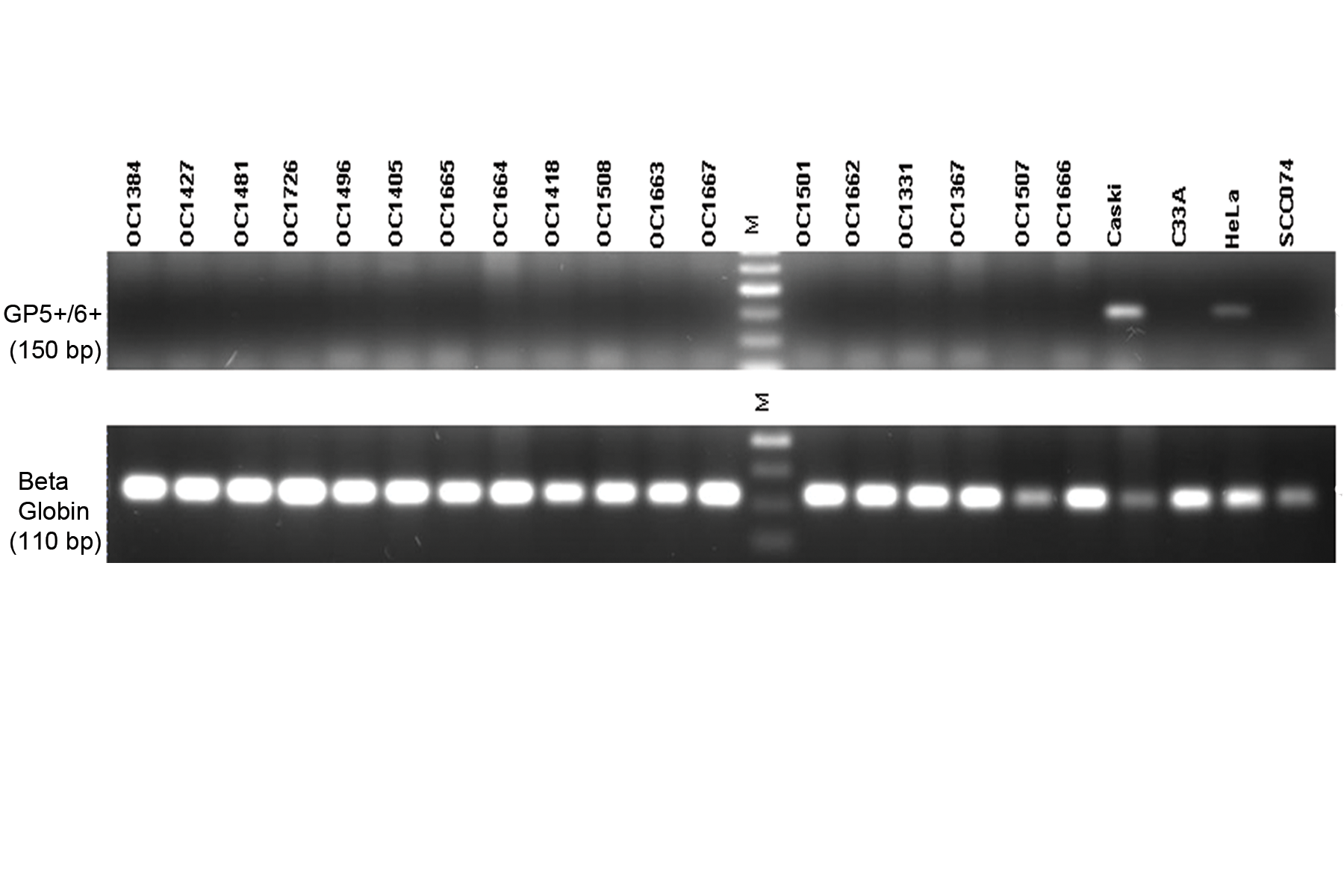

Supplement: Figure S1 — Screening of HPV DNA in tumor samples. Representative gel picture of HPV general primer pair (GP5+/6+) PCR. Beta-globin PCR was done to check the genomic integrity in oral cancer samples. CaSki (HPV-16) and HeLa (HPV-18) cervical cell lines were used as positive controls, while C-33A and SCC074 (HPV negative) cervical and oral cell lines were used as negative controls. M: 50 bp marker. (TIF) [file pone.0017250.s001.tif]

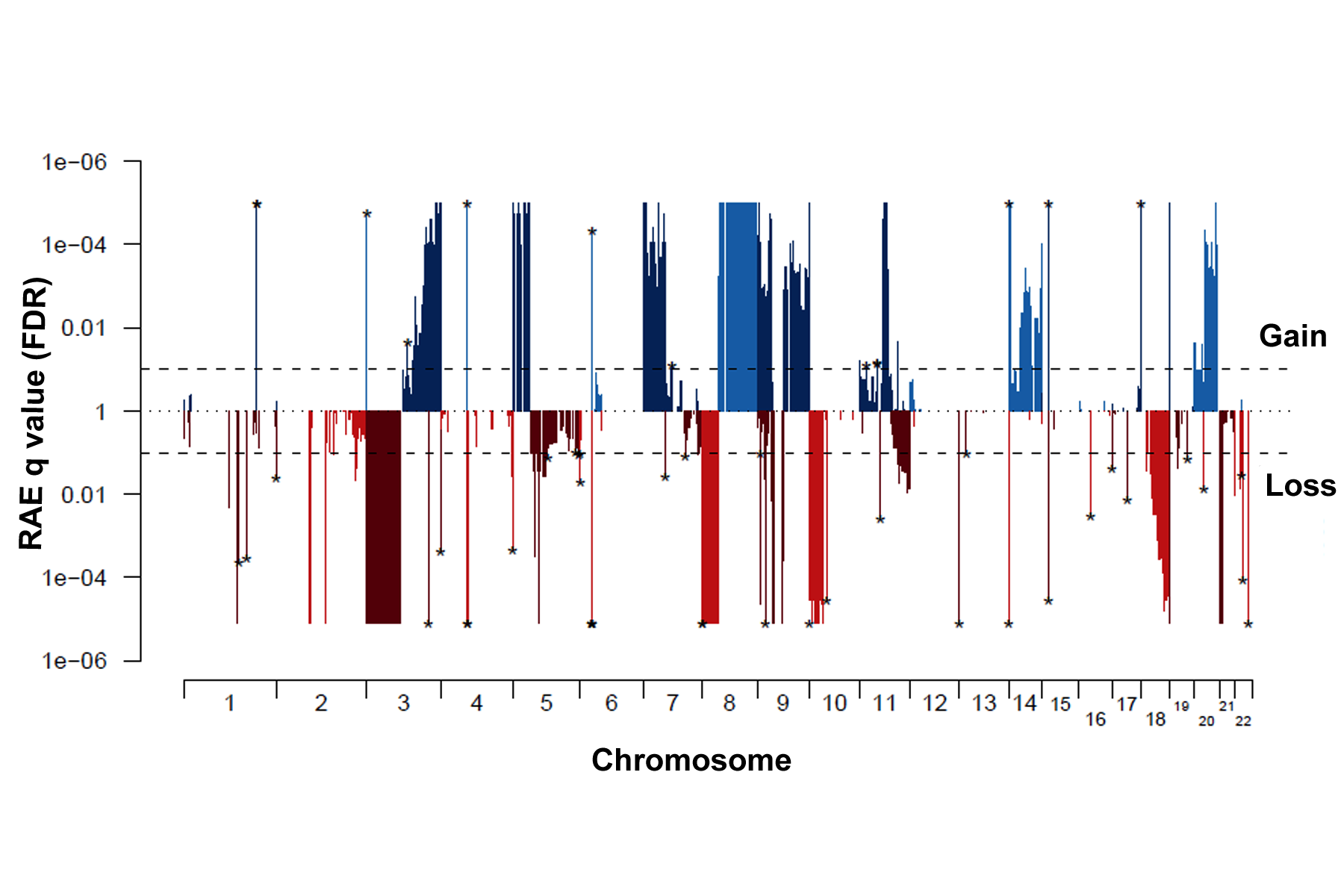

Supplement: Figure S2 — Manhattan plot for statistically significant genomic alterations in OSCC. The false discovery rates (q-value; y-axis) for gains (blue) and losses (red) are plotted against the 22 autosomes (x-axis). The threshold for significance (q≤0.1) is indicated by a dotted line. Stars (*) indicate known copy-number variants (CNVs) according to the database of genomic variants (DGV). (TIF) [file pone.0017250.s002.tif]

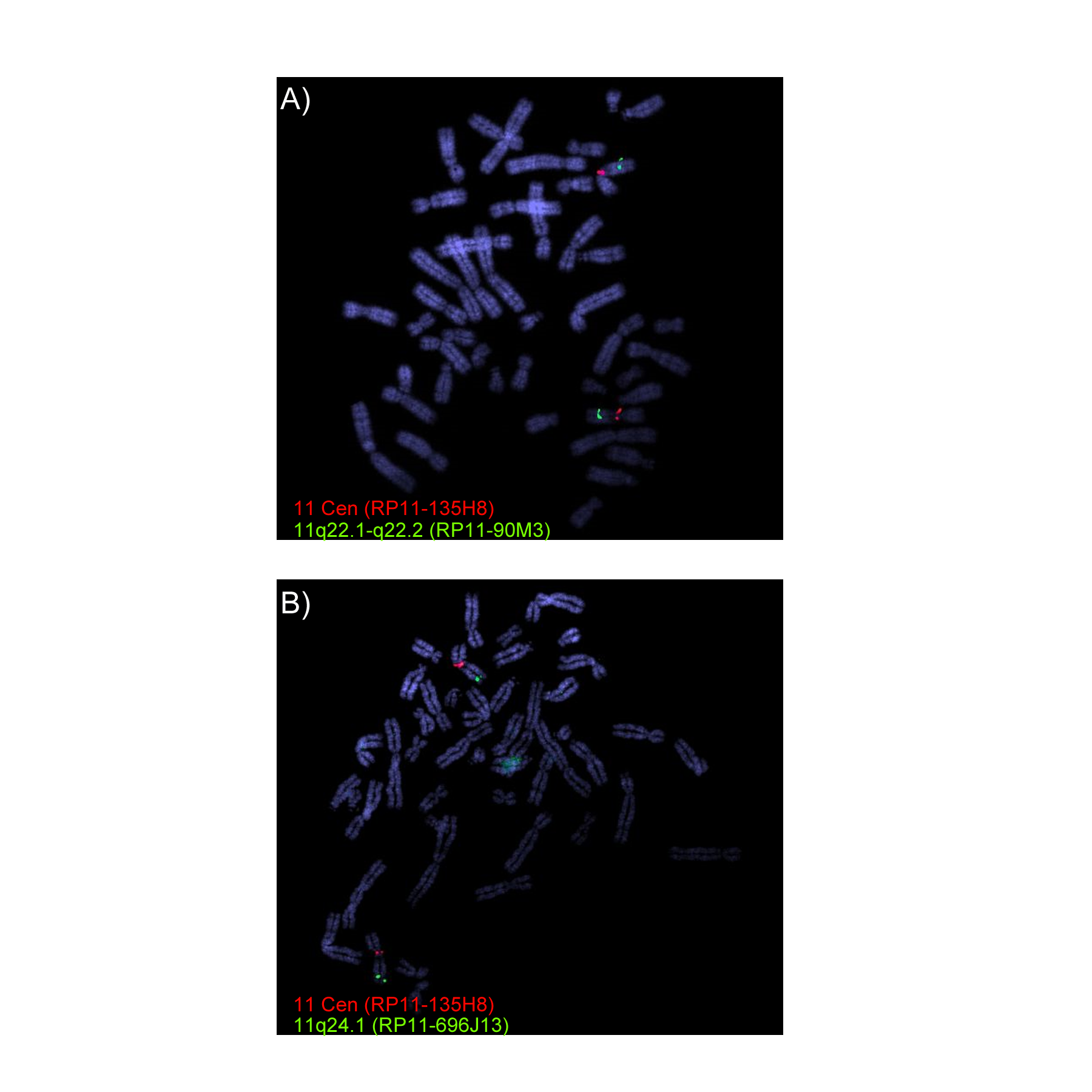

Supplement: Figure S3 — Metaphase FISH confirming the specificity of BAC clones. A representative FISH image of metaphase plates with A) FISH probes confirming the specificity of loci 11q22.1-q22.2 (Green signals), B) 11q24.1 (Green signals) and chromosome 11 centromere (Panels A and B, Red signals). (TIF) [file pone.0017250.s003.tif]

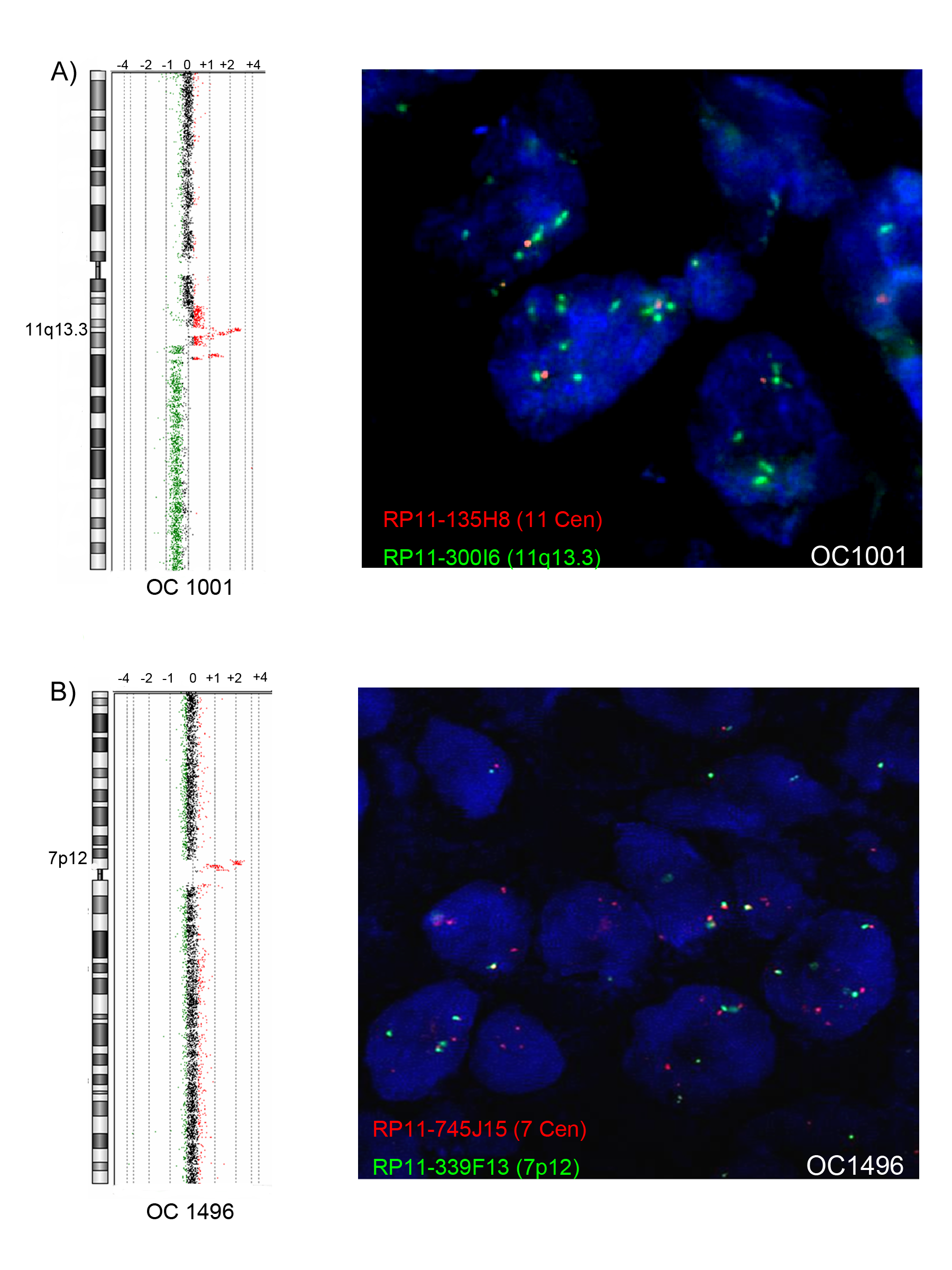

Supplement: Figure S4 — Interphase FISH validating the gain of 11q13.3 and 7p12 revealed by array CGH. A) Validation of 11q13.3 amplification by interphase FISH using centromere-specific (Cy3, Red) and locus-specific (FITC, Green) probe. B) Validation of 7p12 amplification by interphase FISH using centromere-specific (Cy3, Red) and locus-specific (FITC, Green) probe. (TIF) [file pone.0017250.s004.tif]
