# Supplementary material for: Genomic Profiling of Advanced-Stage Oral Cancers Reveals Chromosome 11q Alterations as Markers of Poor Clinical Outcome
Source: PLoS One. 2011 Feb 28;6(2):e17250. doi: 10.1371/journal.pone.0017250 (PMC3046132; doi:10.1371/journal.pone.0017250)
Supplement: Table S1 — Detailed demographic and clinicopathological data for the study group. (DOC) [file pone.0017250.s005.doc]

**Table S1: Demographic and clinicopathological data for the study group**

| **Lab Code** | **Age** | **Gender** | **Site** | **T** | **N** | **M** | **Grade** | **Stage** | **Treatment** | **Recurrence Status** | **Status at Last Follow-up** | **Survival in Months** | **Chewing** | **Smoking** | **Drinking** |
| --- | --- | --- | --- | --- | --- | --- | --- | --- | --- | --- | --- | --- | --- | --- | --- |
| OC1644 | 55 | Female | GBC | T4 | N0 | M0 | Poor | IV | S+CT+RT | Rec | AWD | 13.60 | Current | Never | Never |
| OC1651 | 46 | Male | GBC | T2 | N0 | M0 | Poor | II | S+CT+RT | Rec | DOD | 12.73 | Current | Never | Never |
| OC1647 | 48 | Male | GBC | T4 | N0 | M0 | Moderate | IV | S+RT | Rec | DOD | 18.40 | Current | Current | Never |
| OC1653 | 53 | Male | GBC | T4 | N2 | M0 | Moderate | IV | S+CT+RT | Rec | DOD | 10.43 | Current | Never | Never |
| OC1656 | 56 | Male | GBC | T4 | N0 | M0 | Moderate | IV | S+RT | No Rec | NED | 39.93 | Current | Never | Current |
| OC1645 | 48 | Male | GBC | T3 | N2b | M0 | Moderate | IV | S+CT+RT | Rec | DOD | 15.77 | Current | Never | Never |
| OC1649 | 54 | Female | GBC | T4 | N2 | M0 | Poor | IV | S+CT+RT | Rec | DOD | 7.63 | Current | Never | Never |
| OC1652 | 45 | Female | GBC | T4 | N0 | M0 | Moderate | IV | S+RT | Rec | DOD | 29.33 | Current | Current | Never |
| OC1658 | 39 | Male | GBC | T2 | N0 | M0 | Moderate | II | S+CT+RT | Rec | DOD | 13.67 | Current | Never | Current |
| OC542 | 42 | Female | GBC | T2 | N2b | M0 | Poor | IV | S+RT | No Rec | NED | 50.23 | Current | Never | Never |
| OC1323 | 56 | Female | GBC | T4 | N0 | M0 | Well | IV | S+RT | Rec | DOD | 8.23 | Current | Never | Never |
| OC1747 | 40 | Male | Tongue | T4 | N0 | M0 | Poor | IV | S+CT+RT | No Rec | NED | 22.97 | Current | Current | Current |
| OC0547 | 42 | Male | GBC | T2 | N1 | M0 | Moderate | III | S+RT | Rec | DOD | 51.47 | Current | Never | Never |
| OC1464 | 59 | Male | GBC | T4 | N0 | M0 | Moderate | IV | S+CT+RT | Rec | DOD | 10.07 | Current | Current | Current |
| OC1462 | 49 | Male | GBC | T4 | N0 | M0 | Moderate | IV | S+RT | No Rec | NED | 34.10 | Current | Current | Current |
| OC1440 | 67 | Male | GBC | T4 | N0 | M0 | Moderate | IV | S+RT | No Rec | NED | 32.63 | Current | Never | Never |
| OC1718 | 78 | Female | GBC | T4 | N1 | M0 | Poor | III | S+RT | Rec | DOD | 15.63 | Current | Never | Never |
| OC1487 | 42 | Male | GBC | T4 | N0 | M0 | Moderate | IV | S+RT | No Rec | NED | 28.70 | Current | Never | Never |
| OC1294 | 45 | Male | GBC | T3 | N0 | M0 | Poor | III | S+RT | No Rec | NED | 18.97 | Current | Never | Never |
| OC1719 | 80 | Male | GBC | T4 | N2b | M0 | Moderate | IV | S+RT | LFU | LFU | 5.37 | Current | Current | Never |
| OC1721 | 56 | Male | GBC | T4 | N0 | M0 | Poor | IV | S+RT | No Rec | NED | 33.47 | Never | Current | Never |
| OC1722 | 73 | Male | GBC | T4 | N2c | M0 | Moderate | IV | S+RT | No Rec | DOC | 26.93 | Current | Never | Never |
| OC1726 | 64 | Male | GBC | T4 | N1 | M0 | Poor | IV | S | LFU | LFU | 7.87 | Current | Never | Never |
| OC1750 | 68 | Male | GBC | T4 | N2b | M0 | Moderate | IV | S+RT | Rec | DOD | 4.23 | Current | Never | Never |
| OC1371 | 40 | Male | GBC | T4 | N0 | M0 | Moderate | IV | S+CT+RT | Rec | DOD | 5.97 | Current | Never | Never |
| OC1426 | 44 | Male | GBC | T4 | N2b | M0 | Moderate | IV | S+CT+RT | LFU | LFU | 0.50 | Current | Never | Never |
| OC1405 | 72 | Male | GBC | T4 | N0 | M0 | Moderate | IV | S+RT | No Rec | NED | 26.97 | Current | Never | Never |
| OC1662 | 60 | Male | GBC | T4 | N2b | M0 | Poor | IV | S+RT | Rec | DOD | 16.70 | Current | Never | Never |
| OC1663 | 66 | Male | GBC | T2 | N0 | M0 | Moderate | II | S+RT | No Rec | NED | 36.70 | Current | Never | Never |
| OC1664 | 31 | Male | GBC | T4 | N2b | M0 | Poor | IV | S+RT | No Rec | NED | 39.17 | Current | Never | Never |
| OC1665 | 49 | Male | GBC | T4 | N2 | M0 | Moderate | IV | S+RT | No Rec | NED | 34.57 | Never | Current | Never |
| OC1496 | 37 | Male | GBC | T4 | N0 | M0 | Moderate | IV | S+RT | No Rec | NED | 31.13 | Current | Never | Never |
| OC1507 | 77 | Male | GBC | T4 | N0 | M0 | Moderate | IV | S+RT | LFU | LFU | 7.27 | Current | Current | Never |
| OC1508 | 40 | Male | GBC | T4 | N0 | M0 | Moderate | IV | S+CT+RT | No Rec | NED | 21.73 | Current | Never | Never |
| OC1501 | 50 | Female | GBC | T4 | N0 | M0 | Moderate | IV | S | LFU | LFU | 1.53 | Current | Never | Never |
| OC1418 | 54 | Male | GBC | T4 | N1 | M0 | Moderate | IV | S+RT | No Rec | NED | 24.70 | Current | Never | Never |
| OC1367 | 52 | Male | GBC | T4 | N0 | MX | Poor | IV | S+RT | No Rec | NED | 44.20 | Current | Never | Never |
| OC1331 | 59 | Female | GBC | T4 | N2b | M0 | Moderate | IV | S | No Rec | NED | 43.73 | Current | Never | Never |
| OC1666 | 43 | Male | GBC | T2 | N1 | M0 | Moderate | III | S+RT | No Rec | NED | 51.33 | Current | Current | Never |
| OC1667 | 54 | Female | GBC | T3 | N0 | M0 | Moderate | III | S+RT | LFU | LFU | 0.93 | Current | Never | Never |
| OC187 | 35 | Male | Tongue | T1 | N0 | M0 | Moderate | I | S+RT | Rec | DOD | 15.33 | Current | Never | Never |
| OC105 | 39 | Male | GBC | T1 | N0 | M0 | Poor | I | S | Rec | DOD | 16.67 | Current | Never | Never |
| OC344 | 44 | Male | GBC | T4 | N2 | M0 | Poor | IV | S+RT | Rec | DOD | 9.63 | Current | Current | Current |
| OC519 | 38 | Male | Tongue | T4 | N0 | M0 | Poor | IV | S+RT | No Rec | NED | 81.67 | Current | Never | Current |
| OC398 | 75 | Male | Tongue | T4 | N1 | M0 | Moderate | IV | S+RT | Rec | DOD | 14.50 | Current | Current | Never |
| OC672 | 64 | Female | GBC | T4 | N0 | M0 | Poor | IV | S+RT | No Rec | NED | 67.87 | Current | Never | Never |
| OC739 | 42 | Male | GBC | T4 | N1 | M0 | Poor | IV | S+RT | Rec | DOD | 8.33 | Current | Never | Never |
| OC860 | 70 | Female | GBC | T2 | N2a | M0 | Poor | IV | S | Rec | DOD | 33.10 | Current | Never | Never |
| OC498 | 52 | Male | GBC | T4 | N2c | M0 | Moderate | IV | S+RT | No Rec | NED | 84.10 | Current | Never | Never |
| OC561 | 50 | Female | GBC | T3 | N2b | M0 | Poor | IV | S+RT | Rec | DOD | 5.30 | Current | Never | Never |
| OC588 | 66 | Male | GBC | T4 | N0 | M0 | Well | IV | S+RT | LFU | LFU | 6.03 | Current | Never | Never |
| OC811 | 60 | Male | GBC | T4 | N1 | M0 | Poor | IV | S+RT | Rec | AWD | 13.33 | Current | Never | Never |
| OC939 | 41 | Male | Tongue | T4 | N1 | M0 | Moderate | IV | S+RT | LFU | LFU | 8.07 | Current | Never | Never |
| OC996 | 54 | Male | GBC | T1 | N1 | M0 | Moderate | III | S+RT | Rec | AWD | 35.80 | Current | Never | Never |
| OC849 | 60 | Male | GBC | T4 | N2a | M0 | Moderate | IV | S | LFU | LFU | 0.00 | Current | Never | Never |
| OC1025 | 64 | Male | GBC | T4 | N0 | M0 | Moderate | IV | S+RT | No Rec | NED | 23.73 | Current | Never | Never |
| OC1004 | 56 | Male | GBC | T4 | N1 | M0 | Moderate | IV | S+RT | No Rec | NED | 55.03 | Current | Current | Never |
| OC1001 | 53 | Male | GBC | T4 | N0 | M0 | Moderate | IV | S+RT | No Rec | NED | 65.83 | Current | Never | Never |
| OC1159 | 47 | Male | Tongue | T4 | N1 | M0 | Poor | IV | S+RT | No Rec | NED | 60.47 | Current | Never | Never |
| OC1217 | 40 | Male | Tongue | T4 | N1 | M0 | Poor | IV | S+RT | Rec | DOD | 9.97 | Current | Never | Current |

**Site:** GBC: Gingivo buccal complex (Lower gingivo buccal sulcus, Buccal mucosa, Lower alveolus & Retromolar trigone); Tongue: Laetral border of tongue & Oral tongue.

**Treatment Modality:** Surgery: S, Surgery + Radiotherapy: S+RT, Surgery + Chemotherapy: S+CT, Surgery + Radiotherapy + Chemotherapy: S+CT+RT

**Recurrence Status:** No Recurrence: No Rec, Recurrence: Rec, Lost to follow-up: LFU

**Status at Last Follow-up:** Alive with disease: AWD, Alive and No Evidence of Disease: NED, Dead of disease: DOD, Dead of other cause: DOC, Lost to follow-up: LFU [Lost to follow-up: Patients who did not attend the clinical check-up sessions after primary treatment and as a result their clinical status (recurrence and survival) could not be ascertained.]
