# Supplementary material for: Genomic Profiling of Advanced-Stage Oral Cancers Reveals Chromosome 11q Alterations as Markers of Poor Clinical Outcome
Source: PLoS One. 2011 Feb 28;6(2):e17250. doi: 10.1371/journal.pone.0017250 (PMC3046132; doi:10.1371/journal.pone.0017250)
Supplement: Table S3 — Case-wise chromosomal aberrations at 11q in oral cancer patients. (DOC) [file pone.0017250.s007.doc]

**Table S3: Case-wise chromosomal aberrations at 11q in oral cancer patients.**

**Table S3 A: Gain of 11q13 and loss of distal 11q in OSCC samples.**

| **Cytoband ** | **11q13 Gain / Amplification** | **Distal 11q Loss** |
| --- | --- | --- |
| Case ID |  |  |
| OC1001 | Amplification | No change |
| OC1004 | No change | No change |
| OC1025 | Gain | No change |
| OC105 | Amplification | No change |
| OC1159 | Gain | No change |
| OC1217 | Amplification | Loss |
| OC1294 | No change | No change |
| OC1323 | Gain | Loss |
| OC1331 | Amplification | No change |
| OC1367 | No change | No change |
| OC1371 | No change | No change |
| OC1405 | Gain | No change |
| OC1418 | No change | No change |
| OC1426 | Amplification | Loss |
| OC1440 | No change | No change |
| OC1464 | Amplification | Loss |
| OC1487 | No change | No change |
| OC1496 | Gain | No change |
| OC1501 | No change | No change |
| OC1507 | No change | No change |
| OC1508 | Amplification | No change |
| OC1462 | No change | No change |
| OC1644 | No change | No change |
| OC1645 | Gain | No change |
| OC1647 | No change | No change |
| OC1649 | Gain | Loss |
| OC1651 | No change | No change |
| OC1652 | No change | No change |
| OC1653 | No change | No change |
| OC1656 | No change | No change |
| OC1658 | Amplification | No change |
| OC1662 | Gain | No change |
| OC1663 | Gain | No change |
| OC1664 | Gain | Loss |
| OC1665 | No change | No change |
| OC1666 | No change | No change |
| OC1667 | No change | No change |
| OC1726 | Amplification | No change |
| OC1718 | No change | No change |
| OC1719 | Amplification | No change |
| OC1721 | No change | No change |
| OC1722 | No change | No change |
| OC1747 | No change | No change |
| OC1750 | Amplification | Loss |
| OC187 | Amplification | Loss |
| OC344 | Gain | Loss |
| OC398 | Amplification | Loss |
| OC498 | Gain | No change |
| OC519 | Amplification | Loss |
| OC542 | No change | No change |
| OC547 | Amplification | No change |
| OC561 | Gain | No change |
| OC588 | No change | No change |
| OC672 | No change | No change |
| OC739 | No change | Loss |
| OC811 | Gain | No change |
| OC849 | Amplification | No change |
| OC860 | Gain | No change |
| OC939 | No change | No change |
| OC996 | Gain | No change |

ID: Identification number

**Table S3 B: Summary of 11q alterations in OSCC samples**

| **Event** | **n (%)** |
| --- | --- |
| **11q13 Gains and Amplifications** |  |
| 11q13 gain | 16 (26.6) |
| 11q13 amplification | 16 (26.6) |
| None | 28 (46.7) |
|  |  |
| **Interdependence of 11q13 and distal 11q loss** |  |
| 11q13 gain and Distal 11q loss | 12 (20.0) |
| Distal 11q loss | 1 (1.7) |
| None | 47 (78.3) |
